# "The Hospital" Nursing Mirror

**Published:** 1897-08-14

**Authors:** 


"The Hospital" Nursing Mirror.

Being the Nursing Section of "The Hospital."

[Contributions for this Section of "The Hospital" should be addressed to the Editor, The Hospital, 28 & 29, Southampton Street, Strand, London, W.C., and should have the word "Nursing" plainly written in left-hand top corner of the envelope.]

### News from the Nursing World.

### THE QUEEN'S REPLY.

In reply to an address of congratulation from the women of the United Kingdom and the colonies upon the occasion of the completion of the sixtieth year of her reign, the Queen caused a letter to be sent to Mrs. Cliff Scatcherd, of Morley Hall, Leeds, expressing her pleasure at receiving the address, and stating her belief that "the women of the British race will in the future, as in the past, exercise zealously and faithfully, for the welfare of her people, the powerful influence that by Divine ordering they must ever possess."

### GOVERNMENT BY RULE OF THUMB.

The report anent the transactions of the Prevention of Cruelty to Children is the latest and perhaps the strongest example of the change that has come over the attitude of the public mind with regard to philanthropic enterprise. In the olden time a good motive was looked upon as the only *sine-qua-non* for success. Charity wore so extensive a cloak as to hide not only the sorrows of the sufferers, but all the shortcomings and difficulties that follow only too surely in the train of government by "Rule of Thumb." Nay, there is just cause to believe that these predicaments were courted, rather than arrested, in certain cases, in order that the greatness of the extremity might provoke more liberal benefactions from the hand of pity. We have changed—or are changing—all that. Sound financial systems, strict business principles are rightly demanded in the administration of charities, and before the public gives liberally it likes to make sure that it will get its money's worth.

### NORTHUMBERLAND COUNTY NURSING ASSOCIATION.

A fund is being raised, under the auspices of the Northumberland County Nursing Association, to provide a nurse for the Wooler district. The district is inhabited by a thin, scattered population, and, besides the nurse's salary, heavy incidental expenses must be expected, whilst a bicycle is regarded as a necessary part of her equipment. Lady Tankerville has promised to be president and to give the cycle, and a bazaar is to be held at Chillingham shortly in aid of the fund. Mrs. Rea, of Middleton, will act as secretary and treasurer.

### TRAINING FOR MIDWIVES.

There is much of interest, both to those who are midwives and to those who intend to become so, in the August number of *Nursing Notes*, to be obtained from the Trained Nurses' Club, 12, Buckingham Street, Strand. The club is closed for cleaning purposes until the 16th inst., but on the 19th the next course of lectures for the L.O.S. will begin. The fee for the course of 27 lectures is £3 3s.; fee for nine coaching classes, 15s.; and a single lecture, 2s. 6d. All particulars can be obtained from the secretary, and early applications should be made by those pupils who wish a vacancy reserved for them with a midwife for the practical part of the work. These lectures will be given on Mondays and Thursdays. The attention of all country midwives who are members of a recognised institution is called to the fact that the above useful little journal will be forwarded to them free on receipt of stamped and addressed wrappers.

### THE NURSING DIFFICULTY AT BOURNEMOUTH.

Week after week evidence is forthcoming that it is folly to place the trained nurse of to day under the control of a workhouse master, without hospital training or technical knowledge. So long as this system prevails there can be but little possibility of anything like peace in the workhouse infirmaries. Guardians who have not realised the consequence of such a system are apt to declare, like Mr. Habgood, at a recent meeting of the Bournemouth Town Council, "we are sick and sorry of the whole concern." The master orders a nurse to "lend assistance" in another part of the house; the nurse refuses, the matter is referred to the committee; the nurse resigns, and once more an advertisement is inserted to fill her post, and the whole thing begins over again. In the Bournemouth instance the new nurse is to be engaged under the regulations of the Local Government Board, and the master has signified his intention of "asking the Guardians to define the orders of the Local Government Board for the guidance of the matron, who is superintendent nurse." Such a step, if taken, should lead to the nurses at Bournemouth being placed wholly under the matron.

### BRADFORD NURSES AT THE DUKERIES.

The committees of the Eye and Ear Hospital and the Children's Hospital, Bradford, arranged two trips for the nurses on their staffs. The first, which took place a week before the second, visited the Lake District; the second, consisting of those left at home on the previous occasion, went by train to Retford, and enjoyed a long and romantic drive through Sherwood Forest, inspecting noteworthy places of interest by the way.

### ANNUAL MEETING OF THE ONWARD AND UPWARD ASSOCIATION.

The Onward and Upward Association is a society inaugurated by the Countess of Aberdeen and some friends about sixteen years ago with the idea of bridging over class distinctions, and leaguing all women, but especially mistresses and maid-servants, in a bond of union. There are now 5,000 members divided amongst the 94 branches. The annual meeting was held as usual at Haddo House, and prizes, chiefly appropriate tokens of remembrance of the Queen's long reign, were awarded for various educational competitions, and Lady Aberdeen made a sympathetic and encouraging speech, at the close of which she gave much interesting information about the Victorian Order of Nurses, which she is engaged in organising in Canada.

### HENDON NURSING ASSOCIATION.

Mrs. J. C. Marshall is drawing public attention to the needs of the Hendon Nursing Association. The population has doubled since 1880, and the need of nurses is more than doubled. The nurse now on duty in this district pays upwards of 3,000 visits per annum, which shows that not only her work is wanted by the sick poor, but that it is acceptable to them.

### NEW DUFFERIN MATERNITY HOSPITAL, RANGOON.

Lady Fryer, president of the Burmah Branch of the Countess of Dufferin's Fund, laid the foundation-stone of a new Dufferin Maternity Hospital, Rangoon. In speaking to a large assembly held later in the Jubilee Hall, wherein were gathered many Burmese and Chinese ladies, the Lieutenant-Governor remarked: "I trust that before I cease to hold the appointment of lieutenant-governor you will enable me to see a complete hospital worthy of the province and of its people."

### ST. AGNES DISTRICT NURSING ASSOCIATION.

Mrs. C. Twite presided over the annual meeting of the St. Agnes District Nursing Association, Cornwall. The year has been a successful one both as regards work and finance, the latter being largely helped by the "St. Agnes Boys" in South Africa. Mrs. Twite has resigned the position of hon. secretary, and Mrs. Reedall accepted it. The association has had heavy losses through death this year.

### A NURSE IN A NOVEL.

The public would do well to disabuse its mind of the idea that because a woman is a nurse she is therefore exempt from the trials and failings of humanity. Hospitals and training schools should not be regarded as magic mills in which bad material is ground into good nurses. The fact that this is forgotten, and also because it happens that "The Christian," by Mr. Hall Caine, appears in book form at the commencement of "the silly season," is the reason that the doings and misdoings of the heroine, Miss Glory Quayle, arouse a controversy of which they are not worthy. None but a novel-fed imagination will be deceived for a moment into the belief that any such lack of wholesome discipline is possible in our hospitals to-day, whatever might have been the case formerly, especially in some of the scandalously managed workhouse infirmaries, before the reformation now in progress began.

### A GRUMBLING ASSENT.

Judging by the measure of persecution dealt out occasionally to the Established Church she must be waxing stronger. Notwithstanding the undeniably good work done by the "East London Nursing Society" on unsectarian lines, there was quite a storm in a teacup at a recent meeting of the Whitechapel Guardians on the occasion of £10 being voted to its funds, because the society was "a Church of England institution." Mr. Johnson evidently wished to impose a "religious test," and only yielded a grumbling assent when Mr. Collins bore personal testimony to the good work done by the society. If the members of every creed impose "tests" upon the members of every other form of belief, it seems not improbable that the end of the matter will be a general cancelling of religion altogether, and the only profitable faith will be atheism.

### THE MARY WARDELL CONVALESCENT HOME.

Convalescence from scarlet fever is both trying to the patient and dangerous to the community. For some weeks after apparent recovery infection is possible —therefore the need of such resorts as the Mary Wardell Convalescent Home. It is beautifully situated at Stanmore, Middlesex, and is supplied with every necessary appliance for entertaining the inmates, for disinfecting them thoroughly after the period of probation is over and they are able to return to their usual avocations. The number of patients admitted during the twelve years that have passed since its opening is 2,744. They are drawn from every class, about half coming from their own homes and the remainder from the London Fever Hospitals of the Metropolitan Asylums Board. An appeal, heartily seconded by many friends of good standing, is made now for £2,000 to clear the Home from debt. The expenses are many and heavy, and the cost per patient relatively high—too high, we think—£2 10s. to £3 10s. per week.

### SHIRLEY CHILDREN'S HOSPITAL.

Shirley Children's Hospital is but one of a long list of those institutions which have had to face a falling revenue, with increased work. There is a heavy deficit in this year's accounts, and unless this be remedied, useful as the hospital is, the committee will have no option but to close it. We hope this will not be necessary. The Chairman pointed out that it was absurd to admit and keep patients for an indefinite period on a five-shilling ticket, and a lady subscriber suggested that the dispensary patients might pay something for their medicine. Both points show a more vigorous internal economy to be possible; and until the public are convinced that the money subscribed is well managed, so long will the treasury be empty.

### SHREWSBURY DISTRICT NURSES' HOME.

The general committee formed to carry into effect the foundation of the Shrewsbury District Nurses' Home, which is to celebrate the Queen's long reign, has settled its task in a business-like spirit. The fund collected for the home reaches £1,150. The Mayor has been unanimously chosen first president, and Mr. George B. Lloyd hon. treasurer, and the acting committee until December 31st, 1898, elected. A code of rules for the new association has been drawn up, and the committee requested to secure the advice and co-operation of the medical men in the town. All this is as it should be, and we shall doubtless hear of the district being well and popularly nursed in the near future.

### SHORT ITEMS.

A scheme is now being considered by the sub-committee for providing a cottage hospital for the Denaby and Cadeby Collieries.—The Truro District Nurse Fund is to have a share in the proceeds of the Hospital Saturday Collection. The nurse is most popular and hard-working, making on an average 14 visits a day.—Subscriptions are now on foot for the purpose of providing the Ipswich District Nursing Association with a bicycle; £5 11s. has already been raised. —Subscriptions of about £110 having been received towards the Jubilee Nursing Association at Carrickfergus, it was decided at a meeting of the preliminary committee to invite Miss Dunn, superintendent of the Irish Branch of the "Queen's Nurses," to address a public meeting, at an early date, on the advantages of affiliation with the Jubilee Nursing Association.

### Lectures to Surgical Nurses.

By H. A. Latimer, M.D. (Dunelm), M.R.C.S. of Eng., L.S.A. of London, Consulting Surgeon, Swansea Hospital; President of the Swansea Medical Society; Lecturer and Examiner of the St. John Ambulance Association, &c.

### X.—INFLAMMATION (*continued*)—MODES OF TERMINATION—NURSES' DUTIES IN ATTENDING PATIENTS SUFFERING FROM INFLAMMATION.

Inflammation, then, having been excited by one agent or another, and the local and constitutional effects of it having been manifested in the way I have described, we have to expect some result of the process; and I will now describe to you how the affair may end. Under favourable circumstances and treatment it may terminate by resolution, in which case the escaped cells and fluid will become absorbed, the blood vessels will return to their natural size, and the circulation of the blood within them will return to its natural state; so when we speak of "resolution of an inflammation" we mean that the inflamed part has got quite well again. Under other circumstances, and especially in cases where the process has been of a chronic type, the escaped cells may remain alive in the parts to which they have emigrated, and may be converted into a tough tissue of a fibre-like character; then the inflammation is said to have terminated in fibroid thickening. Another termination of inflammation is by the formation of pus, or matter, and then we are said to be dealing with suppuration and abscess. I daresay you have often wondered where all the matter which arises from an abscess, or from an open wound, comes from. Well, it comes from the blood. Examined under the microscope, matter is found to consist of white blood cells, and it is certain that the greater number of these come from the blood-vessels by emigration through their walls, together with the fluid in which they float, whilst some of them are formed by division of the cells themselves and consequent birth of new ones. When the inflammatory process is very violent, and there is a lack of resisting power in the affected part, ulceration or gangrene are apt to occur, the affected tissues dying in small bits, in the case of ulceration, and in a mass when gangrene has taken place. Both these processes are in the main due to death of the inflamed parts from deprivation of blood; so much pressure is exercised on blood vessels, as well as on the surrounding tissues, by escaped blood cells and fluids, and the circulation of the blood itself in its vessels is stopped so completely, that the parts are starved of blood, and so, necessarily, die. When the surgeon sees such a condition of things impending, he often preserves the life of a region so affected by promptly making free cuts there, and so letting out the compressing fluids.

The inflammatory state is accompanied by constitutional symptoms, the foremost of which are classed under the name of fever. My next lecture will deal with this subject, so I will reserve what I have to say under that heading. I will conclude what I have to say about inflammation by some general remarks on what you, as nurses, will have to do in cases where it occurs. You will have to attend to the local and general results following it. Locally, you will have to see that the affected part is kept in the most comfortable condition possible, and that the appliances ordered by the surgeon in attendance are acting as is desired, and that they are not moved without due reason. An injured part may have been put up in splints or in bandages, and owing to the supervention of inflammation the part may become so swollen that there may be danger of gangrene resulting. In such a case—known by coldness of the limb and lividity of colour beyond the point pressed upon—it will be your duty to send a message to the surgeon that the appliances have become tight, and that the part appears to require fresh attention. Or a patient may be very restless and intolerant of control, and you will have to manage him and prevent him from removing dressings which are applied. Be very careful also in thoroughly removing all discharges from the inflamed area. If the surgeon in attendance has entrusted the daily dressings to you, be sure that you do your work in its entirety. Have the necessary lotion, dressings, and instruments at hand before you disturb the part; let soiled dressings be put in separate basins and taken away at once for destruction; do not disturb the parts you are attending to more than is necessary, and always remember that they are very sensitive, and that rough handling of them is injurious alike to the part itself and to the patient who owns it, as the more pain that is caused during the operation of dressing the wound or inflamed part, the more his nervous system becomes lowered, and the less able he is to resist disease. You will have to attend also to the dieting, and see that his food is taken in right quantities and at the right times. The time was when persons suffering from any form of fever were fed on low diet, with the erroneous idea that by so doing the fever would be starved out or would have nothing to feed itself upon; such treatment was responsible for the loss of many lives. Nowadays we take care to support people suffering in this way, choosing for that purpose foods which require very little effort in the act of digesting them, and which are quickly absorbable. Fevered patients have lost their appetites, and food is often very repugnant to them. By attention to the mouth, in keeping it moist, you will enable them to swallow and to taste, and by bringing to them broths and farinaceous foods in small quantities at a time, and by persuasion, you will get them to take what is ordered. Tact and method go a very long way in these matters, and these are just the qualities which I cannot teach you in lectures, but which you will acquire in the wards during your training here, if you have a real feeling of sympathy for the people under your charge. As for the special treatment of inflammation we shall treat of the same when considering it as it occurs in the various organs which will come under our notice.

### Minor Appointments.

West Ham Union Infirmary.—Miss Katherine Burman was appointed Nurse of the above institution on July 29th. she was trained by the Workhouse Nursing Association at Brownlow Hill, St. Pancras. Miss Burman previously held the position of charge nurse at the City of London Infirmary, and has also been engaged in private nursing. She holds the L.O.S. diploma.

The Sanatorium, St. Helens.—Miss Isabel Kewley was appointed Staff Nurse of the Sanatorium on August 6th. She was trained at Salop Infirmary, Shrewsbury, and has previously been charge nurse of the Fever Hospital, Warrington.

Carnarvonshire and Anglesey Infirmary, Bangor.—Miss Ada W. Paul was appointed Charge Nurse of this institution on July 14th. She was trained at Stroud Hospital, and has since been engaged in private work, institutional and otherwise.

Parochial Hospital, Dundee, N.B.—Miss Robina Ross was appointed Charge Nurse of this hospital on August 2nd. She was trained at the Longmore Hospital, Edinburgh, and subsequently held an appointment at Craigleith Hospital, Edinburgh.

### Post-Graduate Clinics for Nurses.

By a Trained Nurse.

### XXVI.—SOME PRACTICAL HINTS ON MASSAGE.

Having brought the nursing of rest-cure patients to a close, I propose this week, as a suitable conclusion to the subject, to deal with a few practical points connected with massage.

While it is not desirable as a general rule that nurses should combine massage with nursing, a knowledge of its theory and skill in its practice is distinctly the crowning point of a nurse's training, for a great deal of "rubbing" is now employed in surgical cases to relieve and overcome the thickening of joints from inflammatory deposits—in ankylosed conditions and in chronic joint affections. Massage, too, is constantly prescribed in paralysis of limbs, semi-paralysis, and weakness or loss of muscular power. It is found extremely beneficial in the anæmia of dyspepsia, in ovarian and uterine cases, in locomotor ataxia, spinal diseases, nervous exhaustion, and in some forms of rheumatism. In slow convalescence after pneumonia and fevers, and in cases of extreme emaciation, some "rubbing" may be prescribed—of a nature not sufficiently ambitious to call for the special services of a masseuse. Under such circumstances it will be most useful to a nurse if she have some experience in manipulations. In special cases the physician in charge will, of course, see to it that a trained masseuse is employed. But he has a right to expect any trained nurse to understand simple rubbing—as, for instance, the abdominal rubbing now so often employed for the relief of constipation.

Massage—whose origin is lost in antiquity, for its value was recognised in pre-historic times—is coming more and more into favour. It takes the place of the active use by the patient of his own muscles, and this without laying any strain on his nerve centres. After a few days' massage weak and flabby muscles begin to show signs of suppleness and firmness, the secretions of the skin are increased, the masseuse squeezes out the blood in the vessels, sets it to flow at increased speed, thus not only helping to remove effete and waste matters from the blood, but causing a fresh supply of food to the tissues. Nutrition is thus helped enormously.

Another very important point with regard to massage may be mentioned—and this is a point which I have been surprised to find is so often overlooked both in the practice of massage here and in the text-books written on the subject. And this point, which in the United States is the first axiom connected with the science of massage, is that no rubbing, whether partial or complete, should be given during menstruation. The practice has been proved to be distinctly harmful, the patient in such a case frequently suffers from over-fatigue and a sense of exhaustion rather than the pleasant, soothing, and restful effect which should obtain. Added to this, at such a period there is a greatly increased liability to a chill, which may be pelvic or general. And massage in some cases given during the menstrual period will produce menorrhagia—while in others it may result in amenorrhœa.

The masseuse should at all times and for all cases perform her rubbing operations—so far as this is possible—under the blanket in which the patient is wrapped while being rubbed. And, be it remembered, that the patient should wear no clothing while under the ministrations of the masseuse. Delicacy and refinement must at all times form a prominent part in the nursing repertoire, and in this case no law of modesty need be contravened. For it is quite easy to remove the night-gown under cover of the two blankets necessary to envelop the patient who is to be massed. But I would strongly deprecate the giving of massage to a patient who is wearing a linen night-gown. This is a piece of bad nursing—as well as bad massaging—which has constantly come under my notice as the routine practice of otherwise admirable nurses. And the danger of the patient getting a chill under such conditions is so marked that it cannot be too strongly insisted on and widely known that after massage it is most essential that woollen clothing be worn. The surface of the body, heated as it must and should be by the application of massage, is liable to perspiration and moisture, even though this may be insensible and very slight. But it may be quite enough for a linen garment to cause not only a sensation of coldness, but a chill—slight or severe as the case may be. Several garments of flannel or woollen texture—among them a kind of pyjama suit, the legs, bodice, and arms of which untie and take off separately—have been invented as massage robes. And garments of such patterns may be used at the taste and discretion of a nurse who has found them useful in practice.

But in the absence of such complicated combinations, two blankets serve the purpose admirably, and the patient should lie quiet for at least half an hour in the blanket wrap in which she has been rubbed. Then a woollen or flannelette night-dress—or pyjamas, or anything you will, so that it be not cotton or linen—may be substituted. It is not always easy to induce a patient, warm after the passive exercise of massage, to consent to a woollen garb in summer weather. But the nurse must carry this point—and let it be her province to suit the garment to the prevailing temperature, and select wisely the thinnest, least oppressive flannel substance for summer wear that she can choose.

A question constantly asked by nurses is whether any kind of oil or lubricant should be used for the hands of a rubber. Dr. Weir-Mitchell and other massage specialists condemn the practice unhesitatingly. For if massage be properly done there is no danger of chafing the skin. Unquestionably a small quantity of oil on the palm covers a multitude of massage sins. But it is the mark of the amateur. The masseuse who feels the necessity for oil is generally that type who uses her fingers too much. So long as the greater pressure is exercised by the ball of the thumb and the palms, pressure on the patient's skin will be even, and chafing not probable. Massage movements should be rhythmic, for irregular movements are most unpleasant, while a skilful masseuse learns to make equal pressure with both hands. Be careful that the patient's body is well relaxed before rubbing, and it is an excellent plan when giving abdominal massage to place pillows under the knees, so as to thoroughly relax the abdominal walls.

Duration of massage treatment varies from twenty minutes to a full hour. During the first week give only twenty minutes daily, so that the patient will not feel fatigued. Increase five minutes daily to full time, according to the doctor's instructions.

The best time is midway between two meals. But a weak patient or one undergoing full treatment should take a whipped egg or some beef-tea half an hour before massage begins.

Where patients suffer from insomnia half the massage treatment may be ordered at night, since rubbing, well given, generally induces sleep. In chilly patients it is better to rely on deep manipulations, as percussing and surface "flicking," useful in some cases, increase chilly feelings. For chilly patients, so soon as the feet and legs are massed, draw on warm woollen stockings. After full massage the temperature of the body is usually raised as much as a degree or even a degree and a half.

Swedish movements are largely employed in surgical cases, as also in partial loss of power in limbs. For several days the rubber is instructed to slowly and passively flex the limbs. The next stage is to induce the patient to assist these movements, and he goes on to make active movements on his own account. The last stage is reached when the rubber resists the active movements made by the patient.

### The Society for Prevention of Cruelty to Children.

THE result of Lord Herschell's inquiry into the charges of mismanagement against the executive of the S.P.C.C. is now published. This society, which is most deservedly popular, and which has accomplished an enormous amount of good work, has been overtaken by the fate that so often hinders and injures philanthropic enterprise, even when conducted on genuine principles.

Mr. Benjamin Waugh, the founder of the society, has devoted all his energies to build up this admirable institution for the benefit of the children, so that his annual salary of £800 is not excessive. But, unfortunately, since 1894, Mr. Waugh took a glass of brandy and milk every morning, which was ordered by his doctor, at the society's expense. This has given a former official the opportunity to subject the society, its manner of working, and its officers to the fierce glamour of public inquiry. The accuser in this instance is Mr. Hodge, who until a few months ago was chief clerk in his department, and who provoked the present inquiry, although he says he acted from a wish to obtain reforms in the administration of the society. Be his motive what it might, his conduct has been such as to cause Lord Herschell and his colleagues to discredit his evidence. Mr. Waugh, the inquiry proves, has carried on the affairs of his society honourably. But—and were it not for this "but" the accuser might not have had occasion to bring the present charge—he has been somewhat careless for the space of 18 months respecting the payment for his own refreshment.

Apart from this, it is clear from the investigation that has taken place, and it has become clear to many acquainted with the working of the society, that although Mr. Waugh supplies the enthusiasm and secures the utmost sympathy from the public for the work, the administration, both financial and executive, must be placed in other and better hands. It is notorious that men of business, wealth, and repute have retired from the office of treasurer of Mr. Waugh's society, because they have felt that there was no business method in the conduct of its affairs. What is wanted before the public will be justified in giving this society all the funds it needs to adequately pursue its invaluable labours on behalf of the whole community is a reconstruction of the present system of administration. Mr. Benjamin Waugh should be left in the office of director, but his duties should be confined to work outside the office, for which he is specially well qualified. The financial and business management should be placed in the hands of a new officer, who might be given the title of secretary, and placed under the control and guidance of a strong finance committee. No one should be appointed a member of this committee unless he be a man of proved business capacity as well as of high standing with the public. We have reason to know that if this change were to be brought about, as it ought to be brought about with the utmost promptness, gentlemen of wealth might be found to undertake the duties of treasurer, who would attract to the support of the society a strong finance committee, and then, but not till then, will it be placed in funds, because it will thus command and deserve not only the sympathy, but the pecuniary support of the whole community.

The Assistant Secretary, Miss Bolton, is proved to have been in the habit of drinking spirits twice a day—a fact injurious to the reputation of a woman, as well as to her constitution. There were dinners at the Holborn Restaurant on committee days when she was kept late, and an undoubtedly large cab bill, which, in conjunction with her handsome salary, shows she has fared uncommonly well at the society's expense. That she is a woman is no reason why she should not do so, but unfortunately it has been possible, on this account, to malign the relationship in which she stands with regard to her chief. We endorse the recommendation of the report, "That it would tend to improve financial administration, and would be in other respects advantageous, if Miss Bolton were replaced in the office of assistant secretary by a man possessing financial ability and business capacity."

### Appointments.

### MATRONS.

SANATORIUM, BRADFIELD COLLEGE.—Miss Alice Green has been appointed Matron of the Sanatorium Bradfield College. She was trained in the Nightingale Home, St. Thomas's Hospital, afterwards having charge of wards at the York County Hospital, and the General Hospital, Wolverhampton. Besides district and private work Miss Alice Green has also had temporary charge of wards at M.A.B. Fever Hospital, Homerton, and also matron's locum duties, the last being at the Cottage Hospital, Caterham Valley. Until lately she held the post of nurse-matron at Mr. O. H. Bradnack's Preparatory School, Sutherland House, Folkestone.

WOMAN'S HOSPITAL, WOLVERHAMPTON.—Miss Gertrude Thornton was appointed Lady Superintendent of this institution on August 3rd. She was trained at the Royal Hospital for Children and Women, Waterloo Bridge Road, S.E., and took the L O.S. from the City of London Hospital. Miss Thornton held the post of assistant matron for a short time at the Royal Hants County Hospital.

ROYAL EDINBURGH HOSPITAL FOR SICK CHILDREN.—Miss Sara Moore was appointed on August 1st Sister to out-patient department of the above institution. She was trained at Belfast and has previously and successively held the posts of staff nurse Royal Hospital, Belfast, head nurse Deaconess Hospital, Edinburgh, matron Queen Victoria Convalescent Home, Belfast.

FLORENCE NIGHTINGALE HOSPITAL FOR INFECTIOUS DISEASES, COUNTY BOROUGH OF BURY.—Miss H. Hudson was appointed on August 5th Nurse-Matron of this hospital. She was trained at Staffordshire and Bristol Infirmaries, and has held the position of charge nurse at Stockport Union. She has recently been acting matron at Stockport Fever Hospital.

CITY OF LONDON INFIRMARY.—Miss Elizabeth Steuart was appointed on August 10th Matron of the above institution. She was trained at Liverpool Workhouse Infirmary, and has been charge nurse at the same institution. For the last six years she has been assistant matron Mile End Infirmary.

NEW HOSPITAL, FORT WILLIAM.—Miss B. Hawkins has been appointed Matron of the new hospital in connection with the North British Railway near Fort William. Miss Hawkins was trained at the Hull Royal Infirmary, and for the last six years has been on the private staff of the Royal United Hospital, Bath.

### A Free Guide to Lowestoft.

ONE of the principal attractions at the Imperial Institute Yachting and Fisheries Exhibition is the comprehensive exhibit arranged by the town of Lowestoft, illustrating the fishing and other industries connected with that busy seaport on the east coast. With the object of still further drawing attention to the attractions of this flourishing watering-place, the Mayor and Corporation have prepared for gratuitous distribution a well-illustrated souvenir and guide, copies of which, we are informed, may be obtained at the exhibition, or post free on application to the Town Clerk, Town Hall, Lowestoft.

### H.R.H. Princess Christian's Trained Nurses.

The neat uniformed figures of Princess Christian's trained nurses have become to the inhabitants of Windsor and its neighbourhood familiar as the Castle itself, and the visitor wishing to find his or her way to the "Home" at Clarence Villas will be directed thereto with much promptness, and probably an appreciative word concerning "our nurses." Someone has written about the "individuality of front doors," and certainly there is a home-like and welcoming aspect about this particular house, anticipatory of the cordial greeting which awaits those who are interested in the Home and in its work when they are finally ushered into the superintendent's pretty sitting-room, with its two windows overlooking Clarence Road and Trinity Place.

First, then, to describe the Home. It is a good-sized and comfortable house, Nos. 1 and 2, Clarence Villas, thrown together, with plenty of space for the bestowal of boxes, so necessary for a nurses' home, and ample kitchen accommodation. On the ground floor is the superintendent's sitting - room, before mentioned, and also her office, a visitors' room, the general dining-room, and the nurses' sitting-room. The latter is a pleasant room and contains many evidences of the personal interest taken in the Home and its workers by Princess Christian, whose connection with it is by no means nominal. Here there is a sofa, there a bookcase given by the Princess, and on the walls hang more than one portrait of herself—pictures much prized and admired. Many are the visits paid to Clarence Villas by her Royal Highness, and very real and keen is her interest in all that concerns the nurses and their welfare and in the management of the Home. Upstairs the bed-rooms have been divided into cosy cubicles, dainty little rooms, simply and comfortably furnished, and for the most part made very attractive by their respective owners with photographs and personal possessions. Bath-room and lavatory space is amply provided, and on the top floor is set aside a "sick-room," where a temporarily invalided nurse may be isolated and quiet. It was only last year that the nurses moved into the present Home, so that it is still in its first freshness. Much help towards the fittings and plenishings came from the Princess and other kind friends. Needless to say, there is a special corner sacred to the inevitable bicycle, without which the district nurse of to-day is forlorn indeed.

It is now some ten years since the Home was established, and every year has seen it more firmly fixed in the affections of the people of Windsor, and the extension of its borders. The staff now consists of a lady superintendent, with four district and fifteen private nurses working under her, yet still the demand for nurses grows, until the resources of the Home are at times taxed to the uttermost. The district nurses work in the parishes of Windsor, Holy Trinity, Clewer, and Clewer St. Stephen, and there are besides four outlying districts with a resident nurse in each, at Chertsey, Eton, Addlestone, and Egham. The district nurses are in affiliation with the Queen Victoria's Jubilee Institute, and the following excellent report on last year's work was received by the Princess Christian from the Council:—

"The Council have pleasure in informing your Royal Highness that they have received from the inspector her report of the inspection, on December 5th, of the district nurses' work, which is being carried on in Windsor, and they forward the general result of her inspection: (1) Nurses' work: The work of three nurses was inspected, and found excellent. (2) Nurses' equipment: Very neat and in good order. (3) Manner in which the nurses' books are kept: In excellent order. (4) General remarks: We have received a most satisfactory report of the Home and district nursing working work at Windsor and Eton. Her Royal Highness, the president, and committee are to be congratulated on their beautiful Home, and excellent and efficient nurses.—Arthur L. P. Peill, Master of S. Katharine's, President."

This year the Home has suffered a severe loss by the resignation of Miss Simpson, who has been for seven years associated with it, and for nearly six of these its valued and loved superintendent. Her influence has created and maintained a spirit of good fellowship amongst the nurses and a high standard of work, and sorely will her active presence and helpful energy be missed by one and all. In Miss Robin, who was home superintendent at the Edinburgh Royal Infirmary, the Princess has secured a lady who is likely to carry on the work with ability and success.

In every way it is evident that the Home is doing good and useful work and deserves most cordial support. The committee should find no difficulty in obtaining the increased subscriptions for which they are asking, and which are much needed if the sick poor of the Royal borough are to be as well cared for in the future as they have been in the past.

### Our American Letter.

Two questions greatly to the fore in the American nursing world are how best to raise the standard of the trained nurse and the State regulations concerning the qualifications of midwives. The absolutely untrained woman is now rightly relegated to obscurity, but the majority of trained nurses still fall very far short of the ideally perfect. For some places nurses are required to have been trained for at least two years in a general hospital of over 75 beds, but there are medical men who insist more on the method of teaching than on the number of cases to be attended in the schools. And this is a point that deserves attention. The larger hospital gives variety of experience, the smaller enables the probationer to receive more individual teaching. Possibly the position may resolve itself into the quick clever women being trained in the large institutions; and those slower in mental gifts, but suited to their work by the equally important moral qualities, doing themselves and their calling greater justice after working in the small schools. Be that as it may, our cousins across the Atlantic are setting themselves to improve the standard of their trained nurses in a business-like way.

The other equally urgent matter is also the subject of earnest debate. Some doctors are anxious to see properly qualified women licensed, and thus to drive the amateurs from the field, others look to the growth of dispensaries and the employment of their attendant medical officers to remedy the evil, whilst yet a third party see that there is a class above those attending dispensaries who would be pauperised if compelled to relinquish the attendant whom they pay for one they do not, and this party wishes to see midwives licensed and placed under careful supervision. The importance of this matter can be better gauged when it is known that four-fifths of obstetric cases are now attended by women of this profession.

### Novelties for Nurses.

THE "FIN DE SIECLE" COMB.

This is an entirely new invention, being a combination of a double comb, with either one or more branches round which the hair is bound. The hair is gathered together and twisted as for tying, the comb is opened, placed in position, and shut, thus enclosing the locks firmly near the roots. Full directions are given with each comb, which costs 3s., and can be obtained at 236, Oxford Street, or in the Western Arcade, Earl's Court Exhibition.

### Everybody's Opinion.

[Correspondence on all subjects is invited, but we cannot in any way be responsible for the opinions expressed by our correspondents. No communication can be entertained if the name and address of the correspondent is not given, or unless one side of the paper only is written on.]

### MEN AS NURSES.

"One Who s a Graduate" Male Nurse of Charity Hospital, New York City, writes: I am so sorry, on my return home to England, to find nothing is done for men nurses. Why is it? No training schools; no work. In hospitals the pay in New York is £60 a year; asylums, £80; private nursing, from 12s. to £1 per day. After 10 years' work I was elected a member of the New York Academy of Medicine List of Trained Nurses. I want now to get back to New York. If you wish I will write you more particulars. I enclose my card.

### NURSING IN GREECE.

Mrs. F. L. Palli writes: The number of the "Hospital Nursing Mirror" for July 10th has been sent to me, as it contained an account of Greek ladies as Red Cross nurses. This article has naturally interested me very much, containing as it does such a clear and thoroughly truthful statement of their work during the late painful period of the Turco-Greek war, work of which I, with Dr. Mary Kalapouthaki, were the organisers. I should wish to receive your valuable paper thus made known to me regularly, and beg to forward cheque for yearly subscription.

### A QUESTION OF PROPRIETY.

"A Medical Correspondent" writes: I would esteem it a favour if you would give me information concerning the following points. By whom in English hospitals are enemata administered to male patients? If by probationers, is this the usual practice, and how early in their course of instruction? Is this duty ever allotted to persons other than the nurse in the ward? Are difficulties ever met with in the way of objectionable male patients? Through whose hands and what processes does linen soiled during operations pass? I wish to know what is the usual custom in these cases.

### BADGES AGAIN.

"N. D." writes: I beg to endorse "E. R. W.'s" opinion, and would scorn to make myself ridiculous in the eyes of sensible people by wearing a badge. Have spoken to a number of trained nurses, who feel the same contempt for such smallness, whose minds are upon higher matters. Character stamps the individual and needs no outward symbol. Imitation is said to be the sincerest flattery. How about the upper and middle classes, whose latest fashions are imitated both by the unfortunates and their own servants; they belong to a far larger majority than the nursing profession. We hear no complaints from them. Let us be loyal and true, employing all our talents for God's glory and His poor suffering ones—then the matter of uniforms will sink into insignificancy.

### ROYAL BRITISH NURSING ASSOCIATION.

Louisa J. Stacey writes: After the very stormy meeting of the 22nd, at which I was present, I think it behoves all the members to pull themselves together (and if we up to the present have taken little interest in the Association to do so from this time forth) and show the honorary officers that we do appreciate their kindly efforts to keep our Association comfortably going in spite of the cruel reports spread about, and that we do not want a self-constituted spokeswoman—we can, and will, I am sure, when called upon, speak for ourselves (as many of us were prepared to do at the meeting had not so much of the time been taken up by a few who showed so little loyalty to the Association). And I would like to remind those members who think of withdrawing that now (when the Association is supposed to be in difficulty) is not the time to hang back and say they will not pay their subscription, but, on the contrary, to come forward and assist those who are doing so much at this very trying time for us, and not for a moment think "the Association is going to smash." It cannot if we, the members, will it, but must, on the contrary, become a help to us all. As a simple member of the R.B.N.A. I would earnestly say, let us act up to our motto and be "Steadfast and true."

### NARROW-MINDED NURSES.

"Onlooker" writes: In response to "One Who Desires Breadth" may I be allowed to say that to depreciate sympathy and to "encourage any in narrow-mindedness" is the last thing I should wish to do. Perhaps I wrote more from the beginner's standpoint, who, it seems to me, must during her probationership be a "woman of one idea," or she will, unless out of the run of ordinary mortals, find her mind straying back, with maybe a shade of regret, to the pleasures she may have indulged in in her past life. Surely it goes without saying that sympathy and broad-mindedness are essential to every worker, and a woman worker specially, for is it not almost proverbial that a woman's chief difficulty is in seeing both sides to a question, in breaking bonds which bind her to a narrow and circumscribed outlook to things outside the pale of her enthusiasm? And if necessary in the ordinary woman, how much more in the hospital nurse? But surely in her case it is a sympathy which, in regard to outside matters, must often forego the delights of practical knowledge of that with which she sympathises. For I still maintain that the ordinary hard-worked nurse will find it impossible to keep up with the crowd in the rush and whirl of modern life. As regards sympathy with humanity as a whole—and the types of humanity with which the hospital nurse comes in contact are not always of the most heavenly—may I close with these lines of Lowell's:—

"Who heeds not how the lower gusts are working,
Knowing that one sure wind blows on above;
Who sees beneath the foulest faces lurking
One God-built shrine of reverence and love.
Who feels that God and Heaven's great deep are nearer
Him to whose heart his fellow man is nigh;
Who doth not hold his soul's own freedom dearer
Than that of all his brethren, low or high.
Who to the Right can feel himself the truer
For being gently patient with the wrong;
Who sees another in the evil-doer,
And finds in *Love* the heart's blood of his song."

### DISTRICT MIDWIFERY.

Mary E. Ball writes: Although a reader of your paper for years, I am not in the habit of "rushing into print." I cannot, however, allow the letter published this week on "District Midwifery" to pass without a protest. I have had a considerable amount of experience in that work, and as a trained nurse and midwife who has attended between 700 and 800 cases, may be expected to know a little about it. My last post was to start similar work in a mining district in South Wales, where prejudice as regards the trained nurse reigned supreme. One woman said that she could not have me, as she should have to spend £5 on her house before it would be fit for me to go into it. Others could not engage me for fear of what the neighbours might say. In spite of such opposition, the record for a year and nine months was 230 cases. Is your correspondent not aware of the fact that we are unable now to give a certificate for a stillborn child? The trained midwife does not pretend to be a practitioner any more than the student who does his midwifery, sending for help when the case is too intricate for him to attend. Is your correspondent also unaware of the fact that when a doctor is engaged he does not always (often through no fault of his own) arrive in time? Would the trained nurse be able to cope with an emergency better than a trained midwife? There will always be some women who prefer a female to a male attendant in a normal case of labour, and if they cannot have the trained they will go back to the untrained one, and we know the consequences. One patient of mine had an untrained midwife the time previous. After delivery the so-called midwife took the baby down two flights of stairs to wash it, leaving the patient alone. In the meantime another child was born! The trained midwife would have diagnosed the case and stayed with the patient. The accounts I have heard of them have been horrifying, and would make any-one try, like "Obstetrics," to supersede the untrained mid-wife, if possible. I think it only just to myself to state that in the number of cases mentioned, nearly eight hundred, I have not had a fatal one from the confinement. The doctors always recommended the trained midwife in the town I wrote of. Why, if results are more grievous than with the untrained one? One medical man told me that it was better for him to lose a few cases than to be called in to a case of puerperal fever (as is so often the result of the attendance of an untrained midwife) and so risk the lives of his own patients. I should like to ask "Obstetrics" if the fee charged is as low as that for the untrained? They would not easily obtain more at first. It requires some tact to be able to get on well with the people in their own homes; it is something like being all things to all men, like the Apostle, but I mention this with diffidence, as "Obstetrics" may be more adapted to the work than myself, and the cause of her seeming failure may have to be found elsewhere.

### The Book World for Women and Nurses.

District Nursing on a Provident Basis. By Jamieson B. Hurry, M.A., M.D.Cantab. A reprint from the *Lancet*.

*Apropos* of the Queen's decision to devote the women's Jubilee offering of 1887 to the encouragement of the district nursing of the poor in their own homes comes a reprint from the *Lancet* (July 3rd, 1897), in pamphlet form, of an article, "District Nursing on a Provident Basis," by J. B. Hurry, M.A., M.D.Cantab. He seizes the opportunity to ask very pertinently why district nursing should not be organised on a self-supporting basis, and points out one or two ways in which it could be worked, to the advantage of the poor, not only physically but also morally, by keeping alive in them the spirit of self-help and thrift and avoiding the demoralising surrender of individual responsibilities on to the shoulders of too-ready helpers. Free nomination tickets could be supplied to subscribers of a certain amount, which would provide for the nursing of those who, through extreme poverty, could not pay for themselves even the smallest annual subscription. One nurse would be sufficient for a district containing 4,000 members, and the expense would be, roughly speaking, £80 to £100. In spite of the self-supporting theory it is good, though not necessary, to have a capital fund, such as is being raised in many localities, as it provides many very desirable etceteras—for instance, a home for the nurse, various appliances useful and necessary in sick nursing, and at the same time gives the movement a good start. The author is to be congratulated on having put his ideas into reasonable, clear, and readable form.

### Presentations.

Miss M. G. Cowan, who has held the post of Lady Superintendent of the Sunderland Union Infirmary for over four years, and is now leaving to take up a similar position at King's Norton, was presented by the Medical Superintendent, on behalf of the medical and nursing staff, with a silver cake basket as a token of their high esteem and regard. Miss Cowan has also been presented with a beautifully-engraved silver card salver by the master and matron of the workhouse as a mark of their esteem. Miss Cowan is carrying every good wish from those with whom she has worked so harmoniously and successfully.

### Where to Go.

The Chemists and Druggists' Exhibition.—We are informed that nurses in uniform will be admitted free to the Chemists' Exhibition which is to be opened on Monday next at Covent Garden Theatre.

### Notes and Queries.

*After-Care Association.*

(335) (1) Would you kindly give me full particulars of the After-Care Association? (2) Is it likely the association would assist a trained nurse who has been recently discharged from the asylum cured?—*Cryno.*

Write to the Secretary, After-Care Association, Church House, Dean's Yard, Westminster, who will give all information. (2) Yes, it seems a suitable case.

*An Opening for a Nurse.*

(336) Could you advise me where to find a neighbourhood offering a good opening for a private nurse, and where house rent is low? I have worked here for nearly four years under one doctor, and he will be pleased to give good recommendations. I am fully trained in general nursing, in monthly nursing at York Road, and massage at Cruchton Hall.—*Breadwinner.*

The only way would be to obtain introduction to a medical man with sufficiently large practice to provide you with constant employment, as moving is much too great a risk without. It is just possible that a suitable advertisement might help you to hear of what you want.

*Continental Nursing Homes.*

(337) Two nurses wish to join a Continental nursing home. Can you give them any advice how to proceed?—*J. K. Appleton.*

This question covers too wide a field. In the first place it would be as well to decide where the nurses would like to go, and then if the climate and conditions under which they must work will agree with their health. Chapter X. in H. Morten's book (Scientific Press, 28 & 29, Southampton Street, Strand) gives an excellent outline on this subject. The nurses should obtain definite work before leaving England, as there is more than a little difficulty in getting it without the introduction from a known medical man.

*St. Thomas's Training for Nurses.*

(338) Will you kindly tell me what kind of hospital St. Thomas's is? (2) At what age probationers are taken? (3) The price of Honnor Morten's "How to Become a Nurse"?—*L. M.*

We will be pleased to answer when L. M. sends her name and address. This is not for publication, but as a pledge of good faith.

*Lady Roberts's Nursing Fund.*

(339) Will you oblige me by inserting in The Hospital the address of Lady Roberts's Nursing Fund? I have already written to the Indian Office, St. James's Park, and also at Whitehall, given in "How to Become a Nurse," but it appears neither can give me any information.—*A. D. B.*

Possibly the reason you can obtain no information is because Lady Roberts selects her own nurses, and receives no applications from nurses.

*Female Dispensers.*

(340) Would you kindly inform me if there is any society for female dispensers where one could obtain information about them, the salary usually required, &c. Some information is required by the doctor here previous to advertising for a lady dispenser?—*F. I. James.*

There is no society for female dispensers, but there are numerous schools which give ladies instruction in this subject, and from which you will probably gain the information you require. See an article on "How to Become a Dispenser" in The Mirror for April 3rd; also "Notes and Queries" for April 10th. Further information might be obtained from the School for Dispensing at the Hospital for Women and Children, 9, Lupus Street, S.W.

*Nurse's Illness.*

(341) Can you tell me who bears the expense of an infectious illness contracted by a nurse in attendance on a case?—*Zara.*

A private nurse pays her own expenses; it is part of the cost of independence. A nurse attached to an institution is provided for in this contingency.

*"The Hospital" Bed.*

(342) Will you kindly tell me if "The Hospital" bed is only available for sick nurses, or is a nurse allowed to use it for a holiday?—*Marie, Bath.*

"The Hospital" bed is for the sole use of nurses recovering from illness.

*The Baths at Droitwich.*

(343) I should be glad if you could inform me what would be the cost for board, lodging, and brine baths at Droitwich or elsewhere. I am troubled with rheumatism, and think that the baths would relieve me.—*District Nurse.*

Write to Dr. Rhoden, who attends daily at the Baths; he will give full particulars. Lodging accommodation is plentiful in the town.

*L. E. M.*—See answer to 338.

